# A Case Report of a Nerve Sheath Myxoma of the Lower Eyelid in a Young Male

**DOI:** 10.7759/cureus.11747

**Published:** 2020-11-28

**Authors:** Rodney C Guiseppi, Fareed Rajack, Jiali Ma, Keale L. Cade, Ali Ramadan

**Affiliations:** 1 Department of Ophthalmology, Howard University Hospital, Washington, USA; 2 Department of Pathology, Howard University Hospital, Washingtoon, USA; 3 Department of Pathology, Howard University Hospital, Washington, USA

**Keywords:** nerve sheath myxoma, eyelid, neurothekeoma

## Abstract

Myxomas are rare, benign tumors of uncertain etiology. Based on the published data, there have been few reported cases of eyelid nerve sheath myxomas and two cases of orbital myxomas. Eyelid nerve sheath myxomas have a similar presentation to neurothekeoma of the myxoid variety, and historically these two diagnoses have been considered interchangeable. At present, the proper diagnosis can be found through protein immunoreactivity with histological staining. Currently in published literature, there has not been documentation of a family history with eyelid growth in patients diagnosed with nerve sheath myxomas. In this case report, we present a 19-year-old male with a potential third recurrence of nerve sheath myxoma who reports a family history of similar eyelid lesions.

## Introduction

Myxomas are rare, benign tumors of uncertain etiology [1]. Nerve sheath myxomas usually develop in the extremities but rarely can develop on the eyelid with a very high recurrence rate [2, 3]. To the best of our knowledge, there have been few reported cases of eyelid nerve sheath myxomas and two cases of orbital myxomas [4]. When we include neurothekeoma, a similar entity to nerve sheath myxomas, there are only 10 cases involving the eyelid in literature [3]. Eyelid nerve sheath myxomas have a similar presentation to neurothekeoma of the myxoid variety, and historically these two diagnoses have been considered interchangeable [5]. However, today proper diagnosis can be found through protein immunohistochemical staining [2, 5]. Currently in published literature, there has not been documentation of a family history with eyelid growth in patients diagnosed with nerve sheath myxomas. 

## Case presentation

A 19-year-old otherwise healthy male presented with a recurrent left lower eyelid lesion status post multiple excisions. He reported a recurrent growth on the left lower eyelid over the past few months. Of note, the patient also reported that both his mother, aunt, and cousin had similar lesions on their eyelids. The patient denied any history of tobacco or illicit drug use. He complained of blurry vision in the left eye with aesthetic concerns, however, he denied any other ocular complaints. Examination was significant for a left lower lid cystic lesion approximately 5 mm in diameter and decreased tear break up time of the left eye. The rest of his ocular examination was unremarkable. 

**Figure 1:**
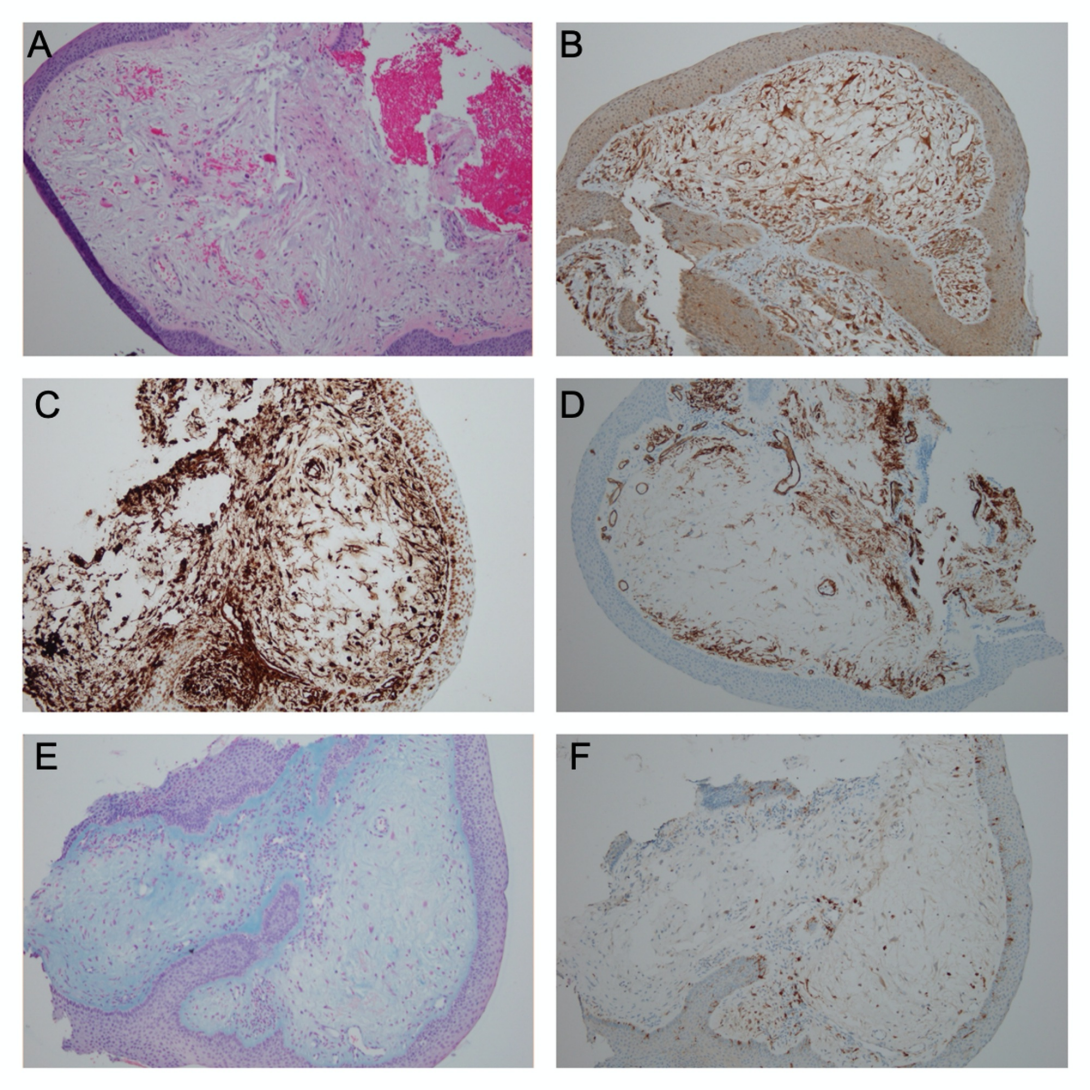
Histopathologic and Immunohistochemical Findings This figure shows the immunohistochemical (IHC) findings at 100X magnification from the biopsy sampled from the patient. (A) H&E stained image benign spindled-stellate and epithelioid cells embedded in a myxoid stroma (B) GFAP IHC stained image is strongly diffuse (C) Vimentin IHC stained image is positive  (D) CD 34 IHC staining revealed positive results (E) Alcian blue stained image is markedly positive highlighting the myxoid stroma (F) S100 IHC stained image shows a focally weak positive.

An excisional biopsy was performed in the clinic without any complications and the tissue was sent for histopathological evaluation. Grossly, the tissue sample consisted of two irregularly shaped, yellow/gray/white masses measuring 0.3 x 0.3 x 0.1 cm in aggregate. Routine hematoxylin and eosin stain was performed which revealed benign spindled-stellate and epithelioid cells embedded in a myxoid stroma (Figure 1A). Based on that information, the differential diagnosis included nerve sheath myxoma, neurothekeoma, and myxoid neurofibroma. Cells were positive for glial fibrillary acidic protein (GFAP) (Figure 1B), vimentin (Figure 1C), CD34 (Figure 1D), Alcian blue (Figure 1E), and S-100 (Figure 1F) but not epithelial membrane antigen (EMA) immunostain. Microphthalmia transcription factor (MiTF) staining was non-contributory because of insufficient tissue sample. The strong expression of GFAP in addition to the positive S-100 stain favored a diagnosis of nerve sheath myxoma. GFAP and S-100 immunostains are usually negative in neurothekeoma. The prominent myxoid change may mimic a cystic lesion which was initially suspected.

His past medical history was significant for left lower lid growth two years prior to this encounter, which was removed. Interestingly, the histopathology report at that time was significant for a gray mass which measured 0.4 x 0.2 x 0.2 cm. Pathology results showed tissue consistent with irritated fibroepithelial polyp of the skin. Periodic acid-Schiff (PAS) stain for fungal microorganisms was negative. Ki-67 did not show an increase in proliferative activity. Further testing may have shown that the lesion may be similar to the one found in this case. We do not have the histopathology report of the previous excisional biopsies that were done. 

## Discussion

Previously in literature, nerve sheath myxomas have been placed in the category of neurothekeoma of the myxoid variant type [5]. In 2005 and 2007 Fetsch et al. showed that the two were separate clinical entities based on immunohistochemical stains [2, 6]. Nerve sheath myxomas are usually superficial, multilobar, highly myxoid masses with a fibrous peripheral border [6]. The lesions consist of Schwann cells which are usually stellate or spindle shaped and also include epithelioid cells. Nerve sheath myxomas are usually strongly positive for S-100 protein and GFAP, however they may also have limited reactivity for EMA and CD34 [6]. The lesions in neurothekeoma are usually non-reactive to S-100 and GFAP and contain more abundant mitotic figures than their nerve sheath myxoma counterpart. [2, 6]. 

Other important differential diagnoses of nerve sheath myxoma include superficial angiomyxoma (cutaneous myxoma) and schwannomas. Cutaneous myxomas are also multilobulated but are usually negative or weakly S-100 positive with positive CD34 and positive actin staining [2, 6]. Schwannomas show low levels of CD34 - positive fibroblasts and EMA - positive perineural cells and may be closely related to nerve sheath myxomas, however further studies are needed to establish this relationship [6]. Given the possibility the patient's family members may have similar lesions, it is important to consider carney complex as a part of our differential diagnoses [7]. Unfortunately, our patient was lost to follow up and the indicated workup for possible carney complex was not done.

In previous literature, it has been noted that nerve sheath myxomas present with a high local recurrence rate after a simple excision procedure [3]. The peak age to diagnose nerve sheath myxoma is within the fourth decade of life, while neurothekeoma often presents in the second decade of life [3, 5]. In addition, neurothekeomas are more common on the face while nerve sheath myxomas rarely occur on the face [2, 6]. Both women and men have almost equal frequency for nerve sheath myxomas while neurothekeoma has a female to male ratio of between 2-4.3:1 [5, 6].

In our case, immunohistological testing played a significant role because our 19- year-old patient had a lesion on his face, which is more typical of a neurotheleoma than a nerve sheath myxoma. In this way, immunohistological staining aids in making the diagnosis of either neurothekeoma or nerve sheath myxoma.

## Conclusions

In conclusion, nerve sheath myxomas are rare tumors which occur even less frequently on the eyelids. Both nerve sheath myxomas and neurothekeomas could be differentiated using the proper immunohistochemical stains. Other important differential diagnoses of nerve sheath myxoma include superficial angiomyxoma (cutaneous myxoma) and schwannomas. This case highlights the importance of obtaining immunohistochemical stains for differentiating nerve sheath myxomas from neurothekeomas. Given the paucity of cases in the literature, more case reports may provide a stronger genetic predisposition, considering that our patient reported a history of multiple close family members with similar eyelid lesions. Because of the high likelihood of recurrence, total excision with clear margins and close follow up is the best treatment for patients.
